# Intra-Subject and Inter-Subject Movement Variability Quantified with Muscle Synergies in Upper-Limb Reaching Movements

**DOI:** 10.3390/biomimetics6040063

**Published:** 2021-10-20

**Authors:** Kunkun Zhao, Zhisheng Zhang, Haiying Wen, Alessandro Scano

**Affiliations:** 1 School of Mechanical Engineering, Southeast University, Nanjing 211189, China; oldbc@seu.edu.cn (Z.Z.); wenhy@seu.edu.cn (H.W.); 2 UOS STIIMA Lecco—Human-Centered, Smart & Safe, Living Environment, Italian National Research Council (CNR), Via Previati 1/E, 23900 Lecco, Italy

**Keywords:** inter-subject, intra-subject, muscle synergies, reaching movements, upper limb, variability

## Abstract

Quantifying movement variability is a crucial aspect for clinical and laboratory investigations in several contexts. However, very few studies have assessed, in detail, the intra-subject variability across movements and the inter-subject variability. Muscle synergies are a valuable method that can be used to assess such variability. In this study, we assess, in detail, intra-subject and inter-subject variability in a scenario based on a comprehensive dataset, including multiple repetitions of multi-directional reaching movements. The results show that muscle synergies are a valuable tool for quantifying variability at the muscle level and reveal that intra-subject variability is lower than inter-subject variability in synergy modules and related temporal coefficients, and both intra-subject and inter-subject similarity are higher than random synergy matching, confirming shared underlying control structures. The study deepens the available knowledge on muscle synergy-based motor function assessment and rehabilitation applications, discussing their applicability to real scenarios.

## 1. Introduction

Quantifying movement variability with detailed assessments is a crucial aspect for clinical and laboratory investigations in several contexts. Variability can be seen at many levels: intra-subject refers to the difference found in the same subject in multiple repetitions of the same task [1]. Inter-subject variability refers instead to the variation of motor patterns when multiple subjects repeat the same motion [2,3]. 

Many studies showed that surface electromyography (EMG) signals contain rich information that could be exploited in many fields. They can be used to decode motion intention for recognizing grasping movements [4] or predicting gait events [5], and, especially, to understand the neurophysiological mechanisms of motor control [6]. It was found that the intra-subject variability is crucial in applications involving patients to track the evolution of the disease for diagnosing and treatment planning [7,8] or to assess healthy people’s performance as a reference for motor function evaluation [9]. Variability is also used to assess motor control in different scenarios for exploring the control mechanism of the central nervous system (CNS) to reduce the dimensionality of muscle control [6,10]. In clinical rehabilitation, inter-subject variability is considered to adapt the design of prosthesis [11,12], and clinical decision-making and rehabilitation [13,14], with the aim to achieve more intuitive and flexible controlling for rehabilitation training and promoting quality of daily living considering intra-subject variability [15,16]. Quantifying inter-subject variability is also relevant for determining ranges of variation when building normative datasets [17,18] used as a reference to assess neurological patients’ motor performances [19,20,21,22]. 

However, especially in comprehensive studies, the intra-subject variability is not assessed in detail, and the data are often averaged across trials to achieve mean patterns without detailed analysis on single trials [23,24,25]. 

Muscle synergy is a method for analyzing motor control strategies by which the CNS simplifies the motor control by activating a limited set of modules rather than each muscle autonomously [26,27,28]. The muscle synergy method has been largely employed in the last decades. Though there are still many issues to debate, the muscle synergy theory has been based on strong basis, including simulations and behavior research in animals [26,28,29,30] and humans [31,32,33,34]. Muscle synergy is currently one of the state-of-the-art methods for studying motor control and appealing for the study of how the CNS deals with motor variability [6]. 

Variability can be analyzed in the study design and set-up. Sources that affect the variability of muscle synergies include trial-by-trial variability, inherent characteristics of subjects, experimental design, motivation, and EMG preprocessing methods [35]. All these factors concur with the variability of muscle synergy intra- and inter-subject. 

Motion variability has already been assessed with muscle synergies. In human locomotor studies, consistency of muscle synergies under different scenarios and intra- and inter-subject were mostly confirmed [1,36,37]. It was found that shared muscle synergies across subjects were highly consistent during walking [1]. Hug et al. determined that despite inter-subject variability of individual EMG patterns observed during pedaling, inter-subject variability showed a similar set of synergies [38]. The same results were found during gait with cerebral palsy and typically developing children [39,40]. However, Kim et al. [41] reported non-neglectable variability of muscle synergies across stride-to-stride gait in children with cerebral palsy. Even if gait and lower limb use are based mainly on stereotyped spinal patterns [32,42], variability was considered in approaches that concatenated trials rather than averaging them to account for intra-subject variations [43]. 

The upper limb shows greater variability because it implements more refined control, more degrees of freedom and flexibility, and motor redundancy [44,45] in respect to the lower limb. Using the time-varying synergy model, d’Avella [31] showed that synergies were intra-subject and inter-subject repeatable when the movements were controlled. Scano et al. [23] analyzed the variability of a large set of upper limb movements across subjects in more variable conditions and showed that inter-subject variability increases in respect to previous works, questioning if not in controlled scenarios, muscle synergies are equally repeatable across subjects as found in some more controlled studies. An analysis of intra-subject variability was not provided, as data were averaged prior to extraction. Pale et al. [6] analyzed the intra- and inter-subject variability of muscle synergies in hand grasps, concluding that intra-subject variability is lower than inter-subject. Moreover, inter-subject variability resulted as not negligible and increasing in inter-session measurements. Low inter-subject synergy variability was instead found when experienced, and new athletes performed a complex motor skill [3], probably because their motor capability is specialized on the analyzed gestures. Delis et al. analyzed various reaching movements in multiple directions to address the nature and functional role of trial-to-trial correlations between synergy activations and found that signal-trial activation of one synergy could affect the activation of other synergies [46,47]. 

Globally, we found that there is limited evidence on the intra-subject variability during upper limb movements that are at the basis of muscle synergy works investigating proximal upper-limb activity. There is also little evidence on how it relates to inter-subject variability: it is an open point if synergies from a subject can generalize through subjects and precisely reconstruct their data according to reconstruction metrics, such as VAF or R^2^. This becomes critical when comparing data from patients (that are typically more variable than healthy individuals) to reference datasets achieved averaging inter-subject data. Should inter-subject data be too variable, synergies could not be really averaged through subjects as they carry subject-specific information, and muscle synergy assessment based on comparisons would have limited reliability [6]. Lastly, very rarely, variability has been assessed in a context of variable, multi-directional, and poorly constrained movements that emphasize these aspects and their translation to real scenarios. Thus, this study quantifies, in detail, the variability of upper limb muscle synergies in intra-subject and inter-subject conditions by exploiting an existing comprehensive upper-limb dataset.

## 2. Materials and Methods

### 2.1. Participants

Twelve young, healthy subjects (3 females, age 25–35 years, weight 69.1 ± 11.5 kg, height 174 ± 8 cm), with no neuro-motor or orthopaedical disease or other motor-related injuries and diseases affecting motor performance, were recruited at the Human Motion Analysis Laboratory, Consiglio Nazionale delle Ricerche (CNR-Italy), UOS Lecco. The study was approved by the CNR Ethical Committee. All participants were informed of the experimental procedure and signed a written informed consent before the experiment. The experiment was conducted under the Declaration of Helsinki. 

### 2.2. Experimental Set-Up

A target board with 8 targets indicated by markers placed on the cardinal points (N, NE, E, SE, S, SW, W, NW) of a circle with a diameter of 0.6 m was designed to direct movements [23]. A marker (“O”) was placed at the center of the circle. A support was designed so that the target board could be freely positioned and oriented with respect to the subjects. A reference marker (“R”) indicating the starting position was located at the subject’s hip height. The reference marker was selected by the subject in a comfortable position but did not interfere with the movement and was at a height lower than the vertical elbow position [23]. 

**Figure 1:**
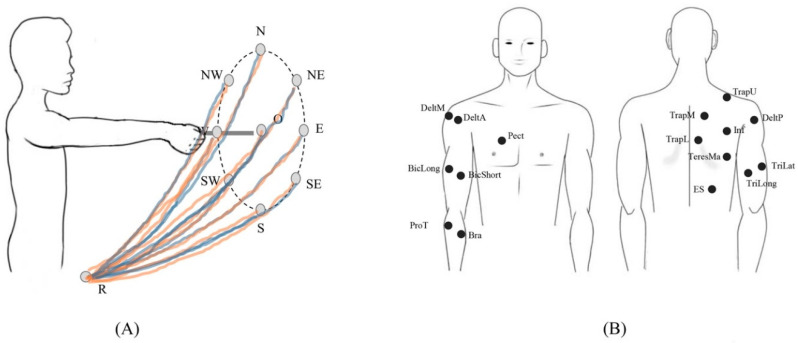
Experiment task (**A**) and positioning of EMG probes (**B**). Sixteen muscles were measured: Erector Spinae (ES), Teres Major (TeresMa), Infraspinatus (Inf), Lower Trapezius (TrapL), Middle Trapezius (TrapM), Upper Trapezius (TrapU), Deltoid Anterior (DeltA), Deltoid Middle (DeltM), Deltoid Posterior (DeltP), Pectoralis (Pect), Triceps Long Head (TriLong), Triceps Lateral Head (TriLat), Biceps Long Head (BicLong), Biceps Short Head (BicShort), Brachioradialis (Bra), and Pronator Teres (ProT).

The experiment consisted of nine point-to-point reaching tasks in the frontal plane from the marker R to each cardinal direction of the target board and back to the marker R (Figure 1A). Subjects started pointing to the first target (O) and went back to R before pointing to the following target. Other targets were reached clockwise (NE, E, SE, S, SW, W, NW, N). There was a one-second pause after reaching each target and about a two-second pause between tasks. Moreover, the trajectory of the movements was not constrained and standardized only for starting and ending points. To account for intra-subject variability and provide reliable analysis, each subject repeated the trial ten times. A trial consisted of nine reaching tasks, one to each target. Each task included the forward phase, one-second pause, and the backward phase. However, due to the gravity, subjects usually returned back to the initial position with low muscle activity and most of EMG phasic activity [23,25] was concentrated in the forward phases. Thus, only forward phases were considered for analysis.

The trials were recorded with a motion capture system (Vicon 8 TVC system, Oxford, United Kingdom) and sixteen surface EMG electrodes (Cometa, Milan, Italy). Before the experiment, subjects wore a set of five markers, positioned on D5 and C7 vertebras, acromion (representing shoulder—S), right elbow epicondyle (E), and styloid process of the ulna (W). Subjects held a 20 cm long pointer identified by two markers (EE1 and EE2). Sixteen muscles’ activities (Figure 1B) were recorded with sixteen surface EMG electrodes positioned according to the SENIAM guidelines [48]: Erector Spinae (ES), Teres Major (TeresMa), Infraspinatus (Inf), Lower Trapezius (TrapL), Middle Trapezius (TrapM), Upper Trapezius (TrapU), Deltoid Anterior (DeltA), Deltoid Middle (DeltM), Deltoid Posterior (DeltP), Pectoralis (Pect), Triceps Long Head (TriLong), Triceps Lateral Head (TriLat), Biceps Long Head (BicLong), Biceps Short Head (BicShort), Brachioradialis (Bra), and Pronator Teres (ProT). Kinematics was recorded at 100 Hz and EMG at 1000 Hz. 

### 2.3. Data Analysis

**Figure 2:**
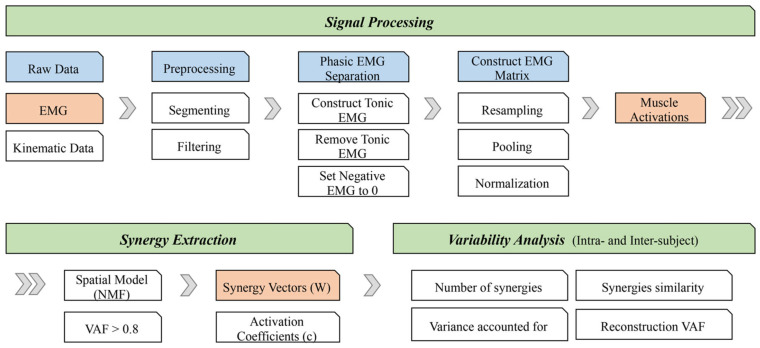
Data analysis flowchart.

Data analysis mainly included three steps: signal processing, synergy extraction, and similarity analysis, as shown in the schematic in Figure 2. 

#### 2.3.1. Signal Processing

**Figure 3:**
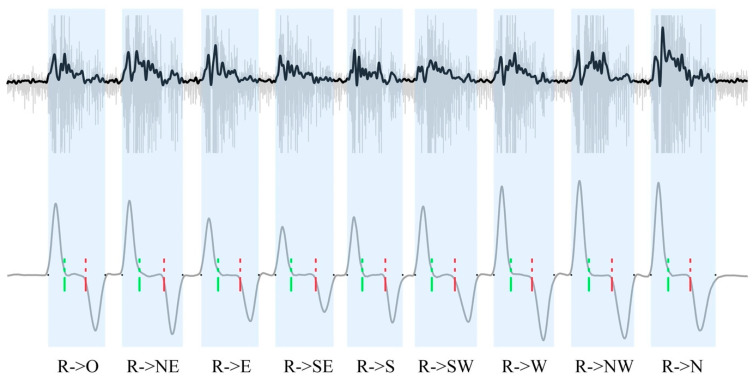
The EMG signal (raw and filtered, upper panel) and velocity profile (lower panel) of a typical subject. The shaded area indicates a full task (reaching and return to the starting point). The green vertical dashed lines indicate the ending time of the forward phase, i.e., the phase from the reference R to the target. The red dashed lines indicate the beginning of the backward phase. A pause lasting one second was performed when reaching the target markers (between each green and red line).

The data were segmented using the kinematic recordings captured with the motion capture system. The trajectory of marker W was computed as the reference for segmentation. Then, the velocity profile of the marker W was obtained and filtered with a smooth moving average filter with a window of 0.2 s to decrease the influence of noise and disturbance (Figure 3) and used to detect the onsets and offsets of the movement. 

Due to the electromechanical delay (EMD) between the onset of the EMG signals and the exerting tension in the muscles, ranging from 10 ms to about 100 ms, with typical values around 50 ms [49], for each trial and forward phase, we extracted EMG signals in the interval (−0.2, 0.2) seconds before the kinematic onset and after the kinematic offset identified with the segmentation based on the velocity profile. This procedure ensured capturing the complete EMG activities related to each movement. All EMG signals were high-pass filtered at 50 Hz (Butterworth filter, 7th order) to remove the artifacts, rectified, and low-pass filtered at 10 Hz (Butterworth filter, 7th order) to obtain the signal envelope [23]. After obtaining the EMG envelope, we further separated the phasic EMG from the tonic EMG following the procedure already adopted in previous works [23,31]. The phasic EMG extracted from each task was resampled at 100 sample points, normalized by the maximum value of each channel across all repetitions of every subject (to allow inter-subject comparison), then pooled together across all tasks. When removing the tonic EMG, some negative phasic EMG could be obtained. We set the negative EMG values to zero [23]. Finally, we got an EMG matrix (muscle activations) with the dimension of 16 (muscles) by 900 (100 sample points × 9 tasks) from each subject and each repetition. 

#### 2.3.2. Synergy Extraction 

According to the muscle synergy method, EMG recordings can be decomposed into two components: synergy vectors and corresponding activation coefficients. In this study, the time-invariant spatial synergy model was used to extract muscle synergies due to its extensive usage in the literature. 

Muscle synergies were extracted using the non-negative matrix factorization (NMF) [50]. The NMF decomposes the EMG matrix M into a linear combination of a set of time-invariant synergy vectors ($w_{i}$) and corresponding time-varying activation coefficients ($c_{i}$) as follows:

$$M\left(t\right)=\overset{N}{\underset{i=1}{\sum }}c_{i}\left(t\right)w_{i}+e\left(t\right)$$

where N is the number of synergies and e(t) is the reconstruction error. The update multiplicative rule was used to compute the synergies (Matlab function nnmf). The NMF requires the number of synergies (i) increasingly from 1 to 16 before the application of the algorithm to be specified. For each i, the iteration was performed 50 times to avoid local minima. For each datum, the variance accounted for (VAF) was calculated to determine the minimum number of N. VAF is defined as 1-SSE/SST, where SSE is the sum of squared residuals of the original signal and reconstruction signal, and SST is the sum of the squared of the original signal [31]. Two criteria were used to determine N: VAF had to be at least 0.8, and the additional synergy had to explain the fraction of the data variation higher than 5% [51]. 

Different numbers of synergies could be extracted across repetitions for each subject. However, to allow purposeful comparisons, in some of our variability analyses, we extracted the same number of synergies for all subjects (see Variability Analysis).

#### 2.3.3. Variability Analysis

The study analyzed the intra- and inter-subject variability under three aspects: synergy modules variability, spatial synergy composition variability, and reconstruction VAF. 

Variability of synergy modules. Synergy module variability was evaluated by two metrics: the number of synergies and corresponding VAF. The coefficient of variation (CV) [6,52] was used to quantify the variability, and significance analysis was also used to quantify intra-subject and inter-subject variability and analyze their difference. 

Synergy variability. We evaluated synergy variability by quantifying the similarity of matched synergies. For intra-subject variability analysis, we first matched the muscle synergies among repetitions of each subject. Then, the cosine similarity of synergy vectors (*SSV*) was calculated as follows:

$$SSV\left(W_{i}^{k},W_{j}^{k}\right)=\frac{W_{i}^{k}\cdot W_{j}^{k}}{\|W_{i}^{k}{\|}_{2}\cdot \|W_{j}^{k}{\|}_{2}}$$

where $W_{i}^{k}$ and $W_{j}^{k}$ are the kth synergy vector from ith and jth repetition, respectively. The range of SSV is 0 to 1. The larger SSV is, the more similar the two vectors are. 

Further, we defined the cosine similarity of the synergy matrix (*SSM*), which is the average of synergy vector similarity of any two synergy vectors from two synergy matrixes, as follows:

$$SSM\left(W_{i},W_{j}\right)=\mathrm{mean}\left(\underset{m}{\sum }\underset{n}{\sum }\frac{W_{i}^{m}\cdot W_{j}^{n}}{\|W_{i}^{m}{\|}_{2}\cdot \|W_{j}^{n}{\|}_{2}}\right)$$

where $W_{i}^{m}$ and $W_{j}^{n}$ are the mth and nth synergy vector of synergies i and j, respectively. 

Inter-subject variability analysis was evaluated by computing the similarity of muscle synergies across subjects. In general, the number of synergies extracted from each subject may vary. However, extracting a different number of synergies may lead to merging and fractionation [7], altering the results of comparisons (some synergies would be denser when fewer modules are extracted, while others would be sparser when more modules are extracted, biasing the results). Consequently, to compute a more reliable inter-subject similarity metric, four synergies were extracted from each subject in this study. This assumption can be justified with many supporting hypotheses. First, the reconstructed VAF across subjects when extracting four synergies was reasonably similar. Moreover, previous studies showed that four synergies are sufficient to account for the variation of the muscle activation in multi-directional reaching movements [51,53]. Our results (see Results) also indicated that four synergies were suitable in the current study. We first averaged the matched synergies across repetitions of each subject. The averaged synergies from each subject were then matched across subjects and computed the SSV and SSM to analyze the inter-subject variability. 

Meanwhile, to verify that the similarity of matched synergies is significantly higher than the random matching, we calculated the random similarity (Rand). First, we constructed a large synergy matrix ($m\times \overset{s}{\underset{i=1}{\sum }}n_{i}\cdot r$, m is the number of muscles, s is the number of subjects, $n_{i}$ is the number of extracted synergies for the ith subject, and r is the repetitions), which consisted of all synergies extracted from all repetitions and all subjects. The random similarity is the average of the similarity of any two paired synergy vectors. 

Reconstruction VAF. To quantify the generalizability ability of muscle synergies to reconstruct muscle activations, the reconstruction VAF (rVAF) was computed as proposed by previous works [6,54]. rVAF is the fraction of reconstructing one muscle activation by fixing the synergy matrix extracted from other muscle activations [51], which assesses how much variation can be reconstructed for other subjects.

$$rVAF=1-\frac{\|M_{i}-W_{j}C_{ij}{\|}_{2}^{2}}{\|M_{i}{\|}_{2}^{2}}$$

where $M_{i}$ is the ith muscle activation, which is from different repetitions of a subject. $W_{j}$ is the synergy matrix from the jth muscle activation (i≠j). $C_{ij}$ is the activation coefficients calculated by the non-negative least-squares minimization algorithm [24,55], where the algorithm factorizes the $M_{i}$ by fixing the $W_{j}$. 

In this study, the reconstruction VAF was analyzed from inter-subject variability. We first averaged the matched synergies across repetitions of each subject. Then, the averaged synergies from each subject were fixed to reconstruct the muscle activations from each repetition and each subject.

#### 2.3.4. Statistical Analysis

Statistical analyses were performed using Matlab (R2020b). A *p*-value lower than 0.05 was considered statistically significant. To analyze the variability of synergy modules among subjects, we first identified the number of synergy modules of each repetition and each subject; then, a non-parametric Kruskal–Wallis test was applied to examine the significance of the modules among repetitions of all subjects. ANOVA post-hoc was used to test the significance of any two subjects. Further, we calculated the coefficient of variation of the number of synergies and VAF across repetitions to analyze the intra- and inter-subject variability of synergy modules. 

To analyze the variability of synergy components of inter-subject and intra-subject, a one-way analysis of variance was used to examine statistical differences. Specifically, we computed the statistical significance of synergy vectors similarity and synergy matrix similarity among subjects (inter-subject) and among repetitions (intra-subject) and compared them to the random similarity. For all the tested datasets, the effect size and statistical power of the employed test were computed through the GPower 3.1.9.6 software (Heinrich Heine University, Dusseldorf, Germany). The results showed that with the enrolled cohort of subjects, we could achieve a statistical power higher than 0.80 and a Cohen’s f effect size above 0.30 (higher than medium) for all outcome measures. 

## 3. Results

### 3.1. Variability of Synergy Modules 

**Figure 4:**
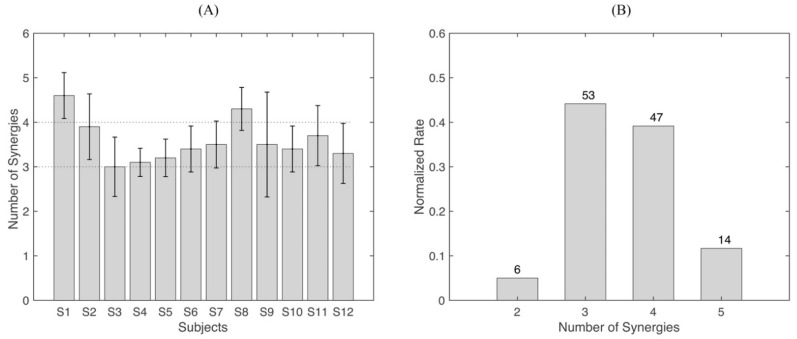
The number of extracted synergies. (**A**) Mean number of synergies across repetitions from each subject. Each bar indicates a subject, and the error bar is the standard deviation across repetitions. (**B**) Number of synergies and percentage from all repetitions and all subjects. In total, 120 synergy extractions were performed in this study (12 subjects, 10 repetitions each).

Figure 4 shows the number of synergies extracted from each subject and the percentage and number of extractions for each number of synergies (two, three, four, and five) in all extractions across repetitions and subjects. Almost all subjects had three to four synergies (Figure 4A). However, some subjects showed five or two synergies in some of their repetitions. If considering the synergies from all repetitions and all subjects, we found that extracting three or four synergies made up a large percentage, 100/120 of the total extractions (Figure 4B). However, the significance analysis shows that there is a significant difference (*p* < 0.001) in the number of synergies across subjects; a further post-hoc test showed statistical differences between S1 (and S8) and other subjects, highlighting significant inter-subject variability in the number of extracted motor modules. 

**Figure 5:**
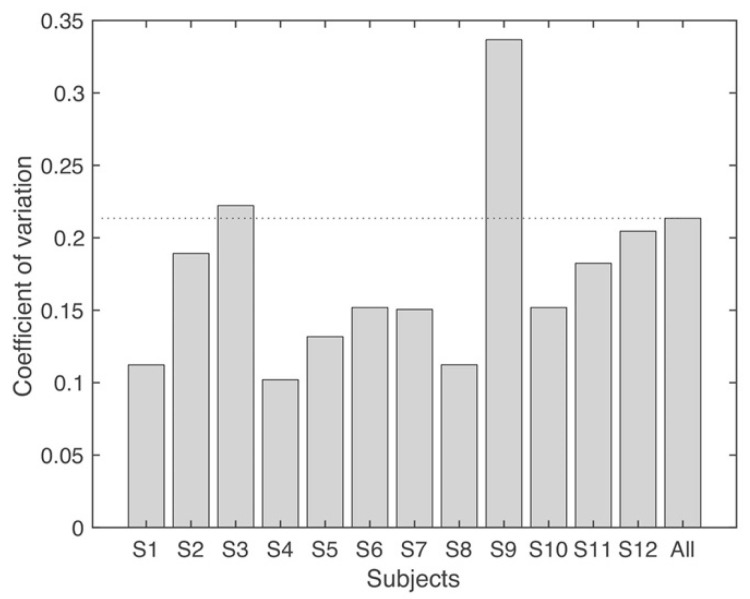
Coefficient of variation (CV) of the number of synergies. “All” indicates that the CV is calculated from all subjects and all repetitions. It includes the variability of intra-subject and inter-subject. The dashed line is the level for “All”.

**Figure 6:**
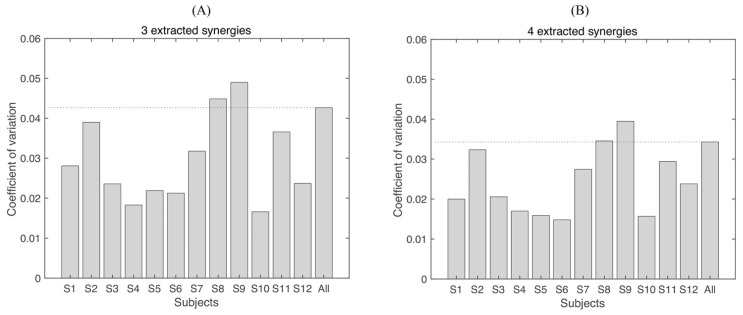
Coefficient of variation (CV) of the VAF. (**A**) Three synergies are extracted (imposing three synergies with NMF), and (**B**) four synergies are extracted (imposing four synergies with NMF). “All” indicates that the CV is calculated from all subjects and all repetitions, which includes the variability of intra-subject and inter-subject. The dashed line is the level at “All”.

The coefficient of variation of the number of synergies and VAF is shown in Figure 5 and Figure 6, for each subject separately and considering all subjects together (“All”). Only 2/12 subjects have a higher CV than “All” in the number of synergies (Figure 5), which means, averagely, that lower variability was found in the number of synergies in intra-subject than inter-subject. When VAF was analyzed, similar results were observed. A total of 2/12 and 2/12 subjects were higher than “All” when three or four synergies were extracted, respectively. 

### 3.2. Synergy Variability

**Figure 7:**
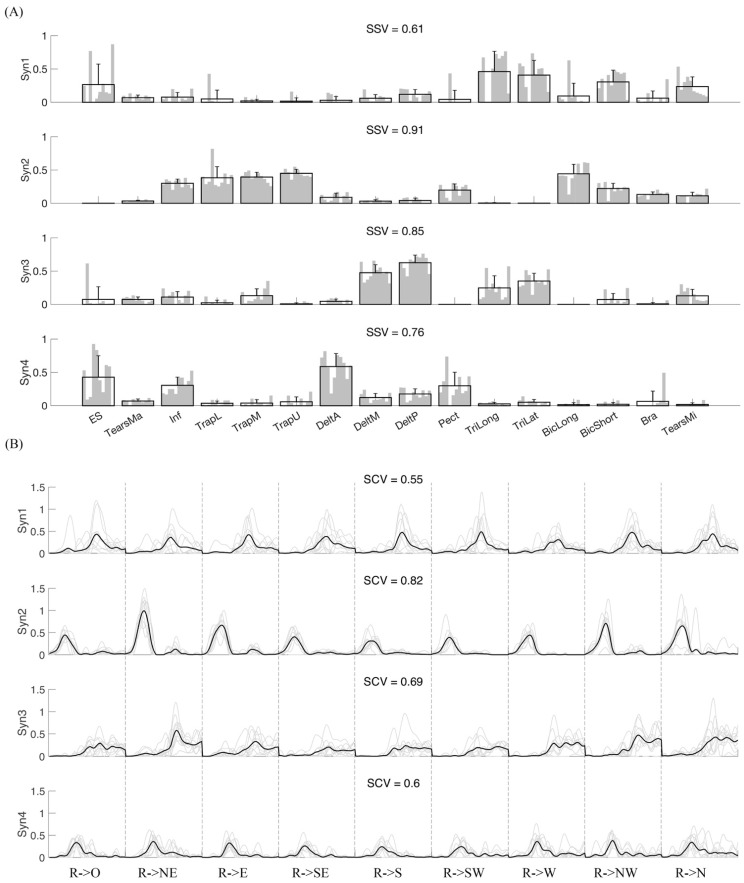
Intra-subject similarity analysis for a representative subject. (**A**) Synergy vectors similarity. Four synergies were extracted from each repetition, then they were matched among repetitions. Each subplot shows corresponding synergy vectors. Gray bars indicate different repetitions, and the black bar for each muscle indicates group mean and standard deviation. SSV is the average similarity of synergy vectors among repetitions. Panel (**B**) shows activation coefficients (AC) similarity. Four ACs corresponding to four synergies were extracted from each repetition. The gray line is the AC from each repetition, and the black line is the mean time course for temporal coefficients across repetitions. SCV is the mean similarity of activation coefficients across repetitions.

**Figure 8:**
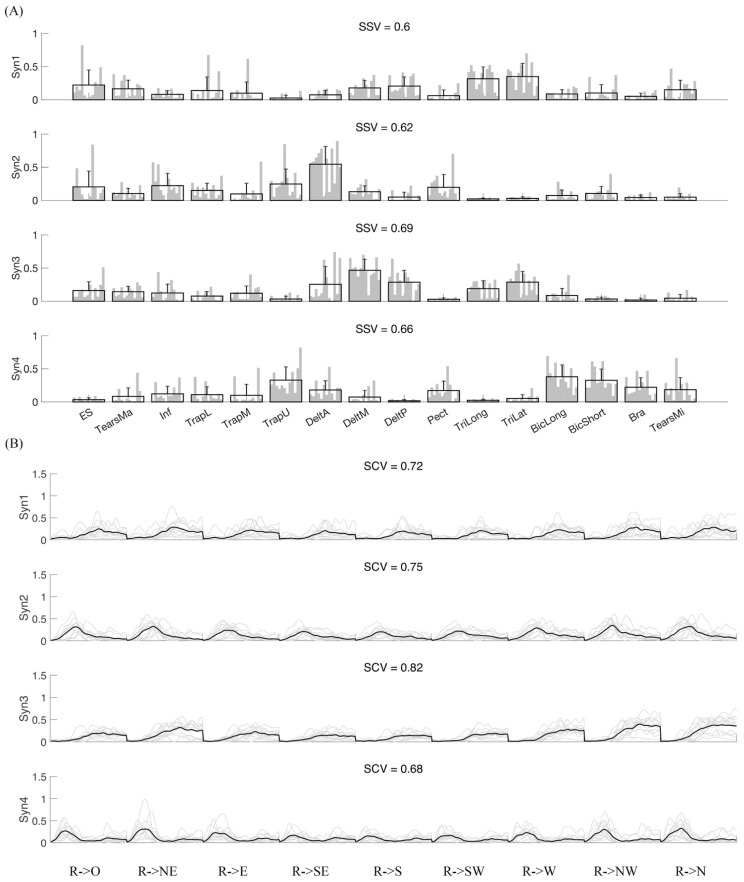
Inter-subject similarity analysis. (**A**) Synergy vectors similarity. Four synergies were extracted from each repetition of each subject. We then averaged the synergies matched across repetitions. Then, we further matched the synergies among subjects. Corresponding activation coefficients were also rearranged. Each subplot shows synergy vectors across subjects. Gray bars indicate different subjects, and the black bar for each muscle indicates group mean and standard deviation. SSV is the average similarity of synergy vectors among subjects. Panel (**B**) shows activation coefficients (AC) similarity. Four ACs corresponding to four synergies were extracted from each repetition. The gray line is the AC from each subject, and the black line is the mean level across subjects. SCV is the mean similarity of activation coefficients across subjects.

Figure 7 shows the synergy vectors similarity and activation coefficients similarity of a representative subject when four synergies were extracted. Four synergies were further extracted from each subject, and the inter-subject similarity is shown in Figure 8. By averaging the synergy vectors similarity of each subject, ANOVA shows that intra-subject similarity is significantly higher than inter-subject (0.79 ± 0.14 vs. 0.64 ± 0.11, *p* < 0.001). 

**Figure 9:**
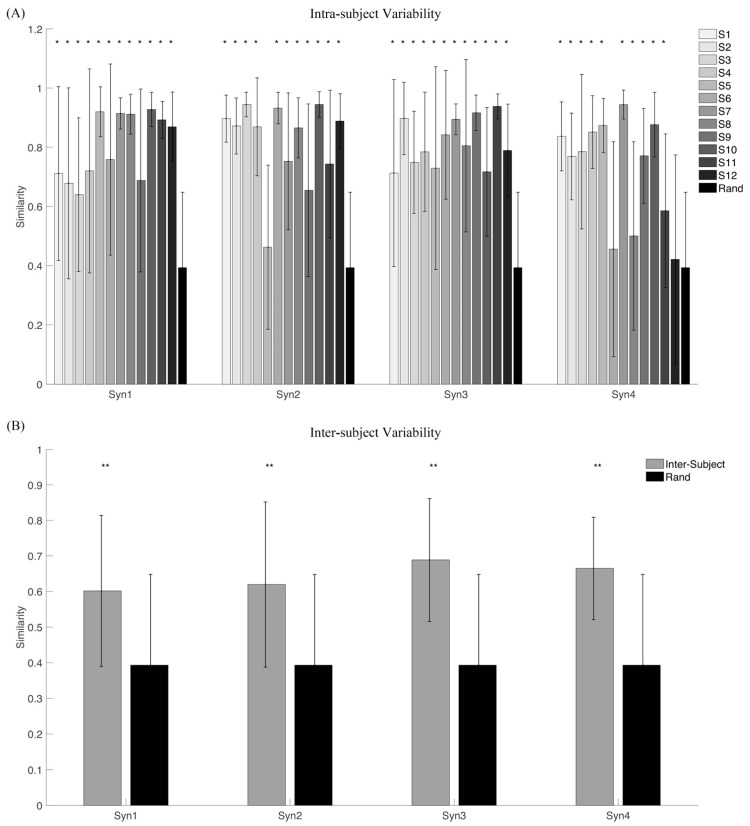
Synergy vectors similarity for intra-subject (**A**) and inter-subject (**B**) conditions. (**A**) Each colored bar indicates the average similarity of synergy vectors across repetitions of each subject. The black line is the standard deviation. Four synergies were extracted for each subject. The final bar, “Rand”, represents matchings for the random similarity. (**B**) The average of the synergy matrix across repetitions is used to analyze the variability of inter-subject. Moreover, the synergies extracted from all repetitions and all subjects are pooled together to compute the random similarity of the synergy matrix. ** indicates significance level is lower than 0.001, and * lower than 0.05.

Further, we compared the synergy vectors similarity of intra- and inter-subject with the random similarity (Figure 9). Almost all synergies from all subjects have a significantly higher similarity level than random similarity with few exceptions (Figure 9A). The first and the third synergy from all subjects are both significantly higher than the random level (*p* < 0.001). The inter-subject similarity level is also significantly higher than the random level (*p* < 0.001) (Figure 9B). 

**Figure 10:**
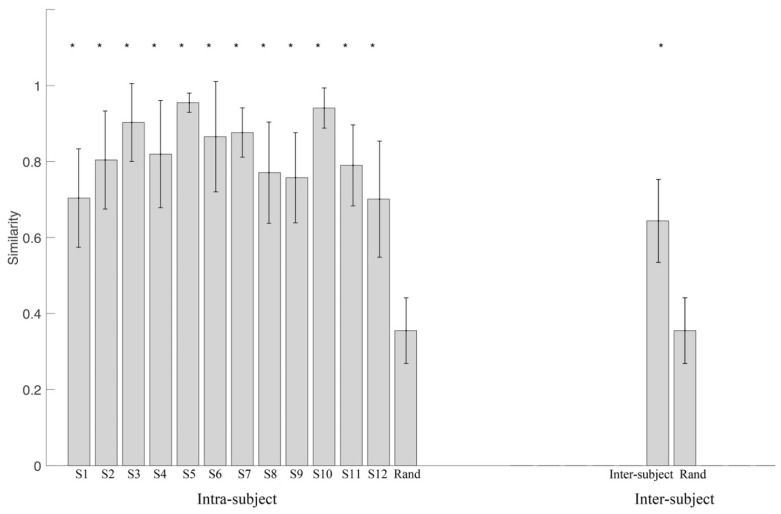
Synergy matrix similarity of intra- and inter-subject. “Rand” is the random synergy similarity. * indicates a significance level lower than 0.001.

The synergy matrix similarity of intra- and inter-subject (in Figure 10) is also significantly higher than the random level. Moreover, we observed that the similarity of intra-subject was higher than inter-subject (0.79 vs. 0.64, p<0.001). 

### 3.3. Reconstruction VAF

**Figure 11:**
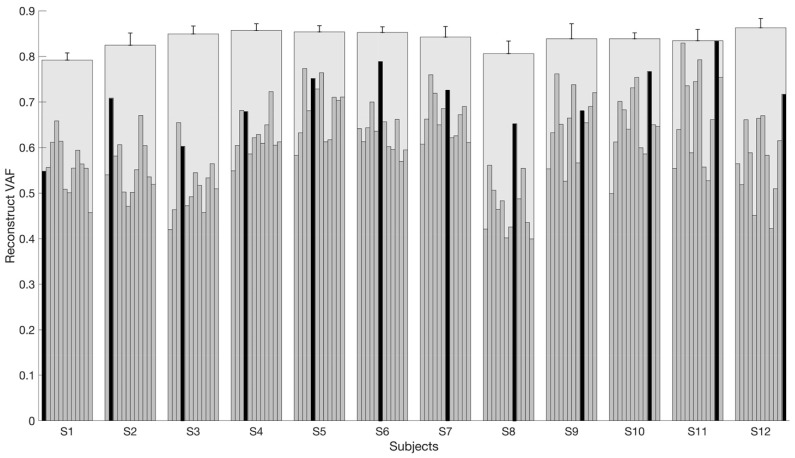
Reconstruction of VAF (rVAF) for the inter-subject condition. Four synergies were extracted from each subject and each repetition. The average synergies across repetitions for each subject were fixed to compute the rVAF of inter-subject (the rVAF that it was possible to reconstruct for each subject by using synergies extracted from other subjects). The thin gray bars indicate the mean rVAF for all the inter-subject possible pairs; meanwhile, the black bar indicates the rVAF that the mean intra-subject muscle activations can be reconstructed using the synergies extracted from the same subject. The wide gray bars are the average VAF across repetitions of each subject (using each task and subject-specific set of synergies).

We reconstructed the muscle activations of all repetitions of all subjects by fixing the mean spatial synergies extracted from each subject. Reconstruction VAF achieved in all subjects is shown in Figure 11. We observed that when the muscle activations were reconstructed using the synergies extracted from the current subject (the black bar in Figure 11), a high rVAF was usually obtained in respect to reconstructions achieved with other subjects’ synergies. On the contrary, when reconstructing muscle activations from other subjects, we often got a lower rVAF (inter-subject rVAF). Figure 11 also shows the mean VAF of each subject across repetitions (blue bars), which shows the variability of intra-subject. As shown, the mean VAF of each subject is all higher than rVAF except for some of the black bars, which indicate the self-reconstruction. 

## 4. Discussion

The current study evaluated the intra- and inter-subject variability in healthy young-adult subjects when performing upper limb reaching movements in a poorly constrained scenario. Variability was quantified by analyzing the number of synergies, the similarity of synergies, and the reconstruction VAF. We concluded that intra-subject variability is lower than inter-subject, and both are higher than the random level. We also concluded that synergies extracted from a subject can only partially reproduce other subjects’ patterns. 

In our study, upper limb reaching movements in the frontal plane were designed. Reaching movements have been largely studied in previous works in healthy subjects [23,24,31] and patients [31,51,56]. Usually, two to five synergies were sufficient to account for the variation of muscle activations in upper limb movements [53,57]. Similar results were found in this study. We extracted two to five synergies from all repetitions. The majority of repetitions extracted three to four synergies, approximately 83.3% of all extractions. This result covered about 92% (11/12) of subjects, and only one subject extracted five synergies when averaging the repetitions. 

The study quantified the variability of synergy modules by analyzing the coefficient of variation of the number of synergies and VAF. As known, CV shows the extent of variability of a set of data in relation to the mean. A large CV-value usually means high variability of the data. In this study, on average, a lower CV was found among intra-subject compared to inter-subject. However, some exceptions appeared in the study (S3, S8, and S9) which showed a higher intra-subject variation in both the number of synergies and VAF. This finding revealed that variability is subject-related and depends on each subject. Comparing with Pale’s work [6], hand grasps variability analysis of intra-session and inter-session, the study found that the CV of the number of synergies varied within 4% for intra-session and up to almost 15% for all subjects, and VAF varied on average less than 3% for intra-session and up to 15% for inter-session analysis. Our results showed a higher CV, about 15% across subjects, when some outliers were excluded for the number of synergies, but a fairly small variation for VAF whenever three or four synergies were extracted (3.1% and 2.5%). On the one hand, different experimental protocols were designed among these studies; on the other hand, data processing methods and the criteria for identifying the number of synergies have an influence on the selection of synergies [35], which both affect the variation of the number of synergies and VAF. The reaching movements were performed under a loose constraint in this study. There might be large variability among repetitions for some subjects, and this set-up may resemble application scenarios where movements cannot be completely constrained. 

Synergy similarity analyses were conducted in both synergy vectors (matched synergy vectors) and synergy matrix (all paired synergy vectors as a whole). In general, the similarity of intra-subject was higher than inter-subject: synergy vectors similarity was 0.80 vs. 0.60, 0.83 vs. 0.62, 0.81 vs. 0.69, and 0.72 vs. 0.66, respectively (Figure 9); synergy matrix similarity was 0.82 vs. 0.64 (Figure 10). Otherwise, all intra- and inter-subject similarities were significantly higher than random similarities. Variability is an inherent property of human movement and was described as “repetition without repetition” by Bernstein in his studies on motor control [58,59]. Variability could account for the redundancy of the musculoskeletal system and contributed to improving the adaptation of humans to complex and changing situations [60]. When applying these concepts to rehabilitative scenarios, patients can achieve some motor function by adopting a compensation strategy that further increases intra-subject and inter-subject variability in respect to healthy people. Our results further revealed that inter-subject variability was higher than intra-subject. This suggests us to select optimal rehabilitation approaches for each subject when synergy-based methods are used for rehabilitation training.

Summarizing our results in the light of previous findings in similar scenarios, they suggest that intra- and inter-subject similarity may be found at a lower extent when movements are not strongly constrained [23]. In fact, d’Avella et al. found that a limited number of time-varying synergies was shared by all subjects, and only a few subjects showed subject-specific muscle patterns [31]. Similarly, in upper-limb multi-directional movements, Delis et al. found that the recruitment of each synergy in every single trial depends crucially on the recruitment of other synergies, which explained the existence of large trial-by-trial interactions between spatial synergy activations [46,47]. However, the higher variability found in this study might also be justified with the slightly higher number of subjects involved in respect to some previous works. Moreover, our recruited subjects were not constrained on pre-defined kinematic trajectories, having only standardized starting and endpoints, with fairly long limb trajectories. This might have promoted motor abundancy [61], which is the possibility to achieve similar dynamic motion outputs with the recruitment of different muscle patterns. It is possible that motor abundancy was found at the intra-subject level, limiting the intra-subject similarity, with further repercussions at the inter-subject level. 

In addition to the synergy similarity analysis, we further evaluated the intra- and inter-subject variability from the perspective of reconstruction VAF. rVAF indicates the generalization ability of the synergies extracted from a subject to reproduce other subjects’ patterns. Israely et al. extracted a set of representative muscle synergies from upper limb reaching movements of different directions by comparing the rVAF across directions [51]. rVAF analysis showed results comparable with the similarity analysis, i.e., the average VAF of intra-subject was on average larger than inter-subject rVAF. In this work, we showed that in poorly constrained scenarios, synergies extracted from subjects have limited power in reconstructing other peoples’ EMGs and muscle generators (lower than intra-subject). This result was already shown in previous studies where movements were more constrained and repeatable [54] in respect to those presented in this study; however, even in that scenario, synergies from the healthy subjects could reconstruct a limited amount of the R^2^ of other subjects. Interestingly, the same effect was not noted on torques that could generalize very well across subjects. While it is clear that some common patterns are shared between subjects (since inter-subject synergy similarity is clearly above random level), they show subject-specific characteristics at the muscle level. These results can be partially explained with effects such as noise in data, subject inaccuracy in performing motion, sub-estimation of the number of synergies due to the limited number of electrodes, or slight subject-specific kinematic alteration that were not neglected in this study. Still, our findings let us conclude that inter-subject variability is higher than intra-subject, and this raises questions about the procedures to adopt for efficient use of muscle synergies in application scenarios.

However, muscle synergies are currently one of the state-of-the-art methods for studying motor control in laboratory and clinical scenarios. Despite needing time-consuming procedures when compared with traditional clinical scales, muscle synergies have been used in a variety of applications [62,63,64,65], especially the rehabilitation field, as a physiological marker to discriminate abnormal motor patterns [7,34,66,67,68]. For example, the studies showed that impaired motor performance (e.g., velocity, accuracy, and smoothness) was related to the changes of muscle synergy in the number of synergies [32,66,67], synergy compositions [7,34,65,68,69], and synergy activations [7,34,65,68], and the alterations of muscle synergy were related to the conventional clinical metrics, such as the Fugl–Meyer scale [65,68] and Brunnstrom stage [69]. Our results show that caution has to be taken when muscle synergies are used for the assessment to build reference databases to evaluate motor control and neurological diseases, especially when movements cannot be fully constrained. Even though a higher similarity of synergies was found in intra-subject, the inter-subject sources of variability cannot be neglected. This becomes even more critical, especially due to intrinsic characteristic changes caused by injury or disease that may lower even more the generalization of muscle generators across subjects. These findings also follow recent works that showed how variability found in inter-subject and inter-session recordings might affect the interpretation of the results, particularly when applied to medical and rehabilitative scenarios. Moreover, there is a growing body of literature that considers single-trial variability as a biomarker that carries information for cognitive aspects that may influence movement. Inter-subject variability can be interpreted in the future also under this perspective. 

Despite these findings to be considered, we noticed that the potential of the synergy-based personalized methods has been proposed in rehabilitation training, for example, functional electrical stimulation (FES) [70,71]. It has reported promising results in improving motor performance and changing the composition of muscle synergy towards those of healthy subjects. 

The results presented in this analysis refer to the employed setup, movements, and analysis pipelines. However, these results cannot be generalized to all scenarios. The methods used for muscle synergy analysis, including the number of muscles [72], adopted muscle synergy model, synergies extraction methods, EMG preprocessing methods [35], and the criteria for determining the number of synergies [70], can influence the outputs. However, previous large synergy-related works followed similar procedures and similar set-ups for muscle synergy analysis, as we did in this study, and the time-invariant synergy model is the most used method to extract muscle synergies. Several studies compared the synergy extraction methods, and they reported that NMF might be the best method for synergy extraction [53,57,73,74].

Lastly, in this paper, we have not considered the effects of factors such as age and sex on muscle synergies in intra-subject and inter-subject variability, as in this study, tested participants were not divided into subgroups. Further analysis is needed when subjects are divided into sub-groups to evaluate whether these factors may influence the results. According to two recent studies [75,76], assessing the influence of sex and age on locomotion muscle synergies, it was reported that muscle control structures are shared between sexes. However, the study revealed the existence of small but defined sex-specific differences in the way humans control locomotion and that these strategies are not entirely maintained in older age. How these effects translate to the complex control of the upper limb is still an open point. Otherwise, large muscle synergy-related studies seldom considered gender influences and age effects.

## 5. Conclusions

This study assessed the variability of muscle synergies intra-subject and inter-subject in a group of healthy young-adult subjects. The results showed that synergy modules and synergy components were less variable among intra-subject compared to inter-subject. Synergy similarity analysis showed that both inter- and intra-subject similarities were significantly higher than the random level. 

## Data Availability

The data presented in this study are available from corresponding author.
